# Supplementary material for: Lignosulfonate Rapidly Inactivates Human Immunodeficiency and Herpes Simplex Viruses
Source: Medicines (Basel). 2021 Oct 3;8(10):56. doi: 10.3390/medicines8100056 (PMC8538131; doi:10.3390/medicines8100056)
Supplement: Supplementary file 1 [file medicines-08-00056-s001.zip › medicines-1371605-supplementary correct.pdf]

Supplementary Materials: Lignosulfonate Rapidly Inactivates Human Immunodeficiency and Herpes Simplex Viruses

Kunihiko Fukuchi, Takuro Koshikawa, Daisuke Asai, Megumi Inomata, Hiroshi Sakagami, Hiromu Takemura, Taisei Kanamoto, Hikaru Aimi and Yuji Kikkawa

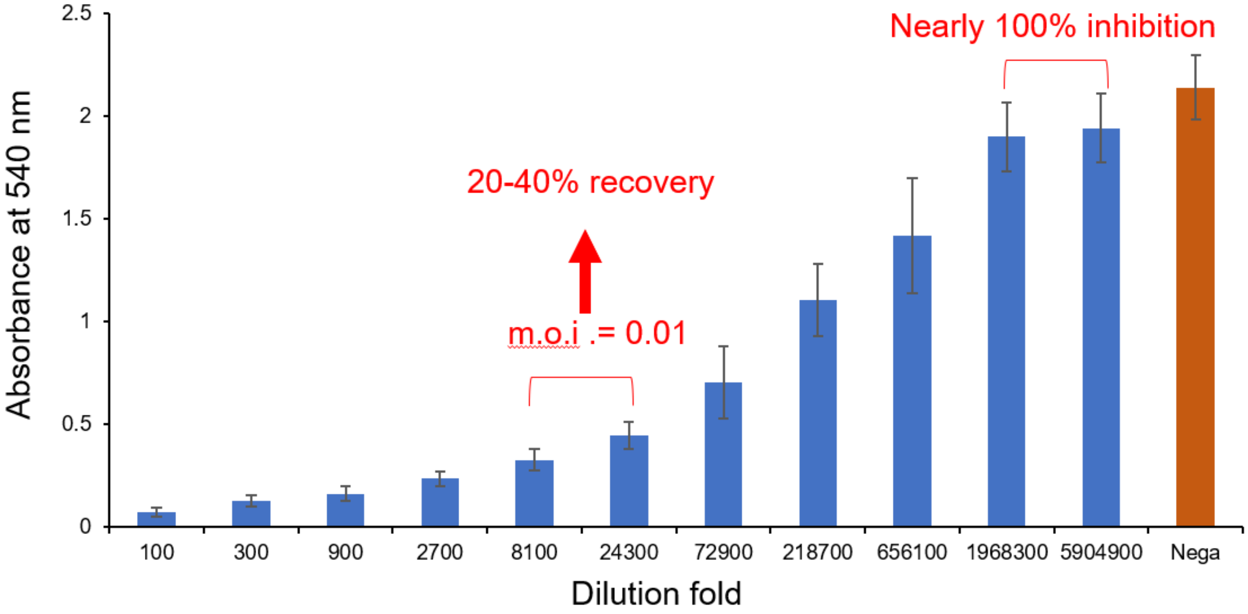

Figure S1. Determination of optimal dilution fold to achieve quantifiable anti-HSV activity. Vero cells (1×10^4 cells) were inoculated with HSV diluted at 100×3^n-fold and the viable cell number was determined by MTT method. Each value represents mean±S.D. of 8 determinants.

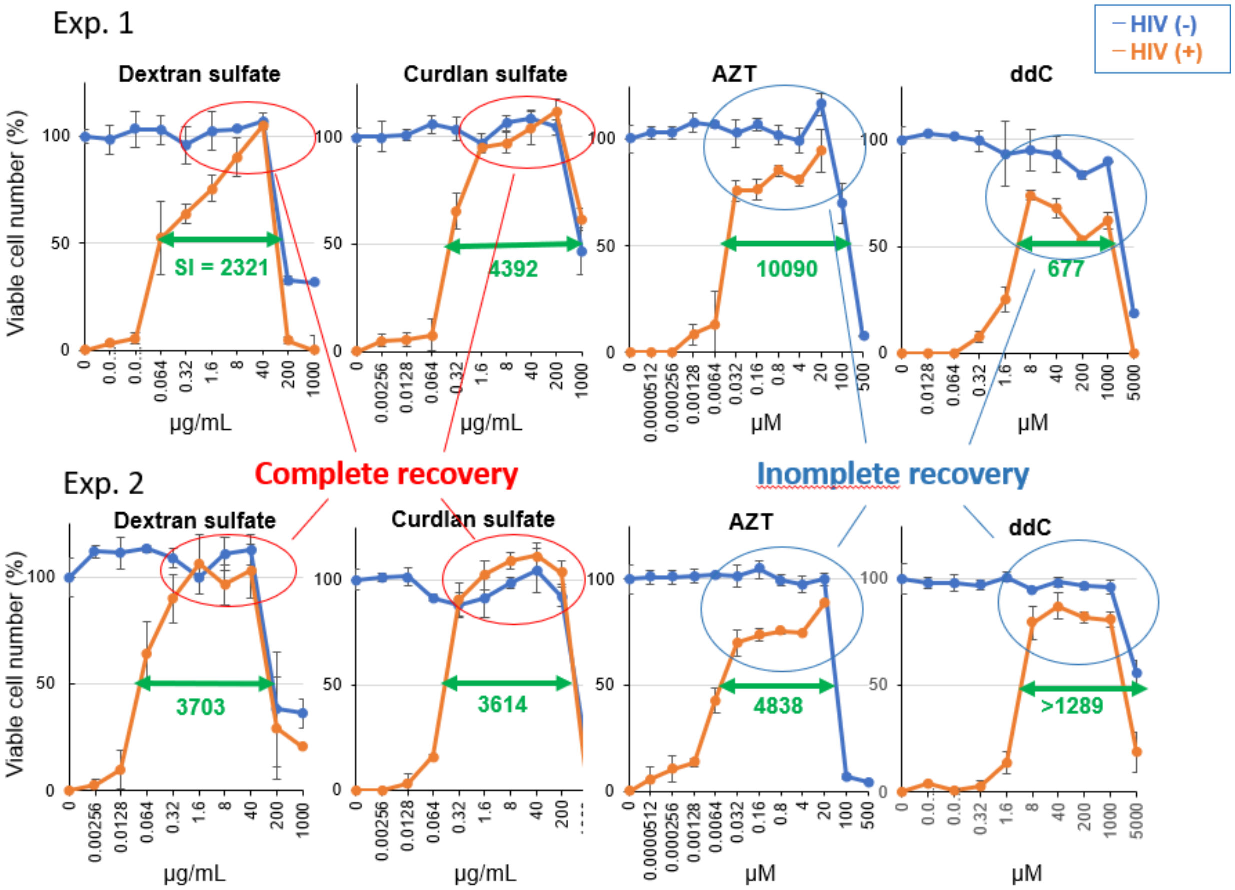

Figure S2. Sulfated glucan achieved higher recovery than reverse transcriptase inhibitors.

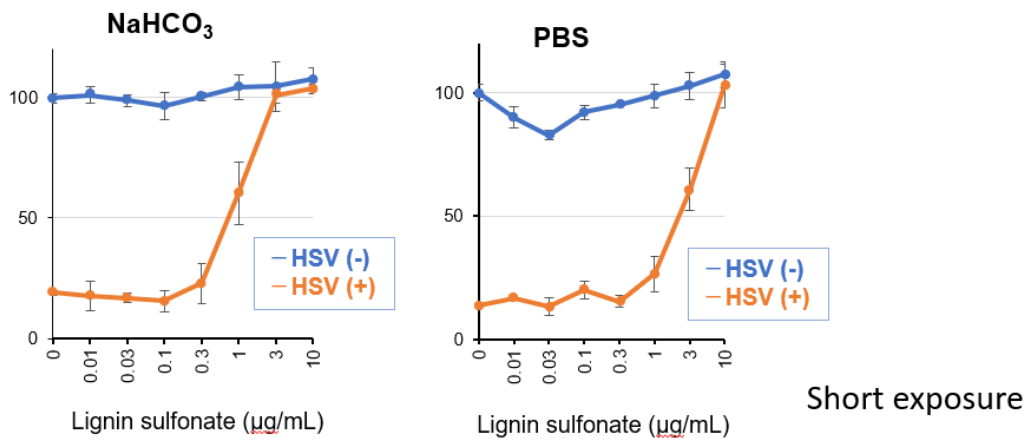

	Solvent	Viability of HSV-infected cells (%)	CC_{50} (mg/mL)	EC_{50}-I (mg/mL)	EC_{50}-II (mg/mL)	SI -I	SI -II	Max recovery (%)
Lignin sulfonate	$NaHCO_3$	19.1	>10	1	0.74	>10	>13.5	103
Lignin sulfonate	PBS	13.6	>10	2.7	2.2	>3.7	>4.5	102

Figure S3. Commercial lignosulfonate showed slightly lower anti-HSV activity (SI ≥ 3.7, >4.5) when dissolved in PBS (as shown in supplementary Figure S1), as compared with lignosulfonate B (SI = 14.1, >21.7) and lignosulfonate E (SI ≥ 16.1, >21.7) (Table 2).

Table S1. Variability of anti-HIV activity (SI value) of four positive controls reported in our 26 papers.

Anti-HIV Activity (SI)					
**Dextran Sulfate**	**Curdlan Sulfate**	**AZT**	**ddC**	**Samples Tested**	**Cited From:**
25323	964	2550	778	Lignosulfonate	This study (Table 2)
2321	4392	10090	677	Lignosulfonate	This study (Table 2)
3703	3614	4838	1289	Lignosulfonate	This study (Table 2)
4200	5294	5082	1340	Pyoktanin Blue	Medicines (Basel). 2021 Jun 22;8(7):33.
2956	11718	23261	2974	Review	Medicines (Basel). 2018 Dec 25;6(1):4.
29485		5082	1913	SE	Medicines (Basel). 2020 Oct 6;7(10):64.
	7142	5624	3868	Mastic extracts	In Vivo 2017, 31, 591-598
300	5805	2017	1670	Chalcones	Anticancer Res 2017, 37, 1091-1098
47619	10204	22893	11898	SE	In Vivo 2016;30(4), 421-426
1935	6028	10403	1916	Oleoylamides	Anticancer Res 2015, 35, 5299-5307
17125	7002	20421	2445	3-Styrylchromones	Anticancer Res 2014, 34, 5405-5411
12363	5523	15882	1789	piperic acid amides	Anticancer Res 2014, 34, 4877-4884
10668	8235	19778	3431	Licorice Root	In Vivo 2014, 28, 785-794
211	3471	20132	2813	SE	In Vivo 2013, 27, 275-284
		17850		Kampo Medicines	In Vivo 2012, 26, 1007-1014
160	781	6931	905	SE-10	In Vivo 2012, 26, 411-418
1378	5606	17746	5123	OTC	In Vivo 2012, 26, 259-264
137919	133305			Luteolin Glycosides and Tricin	In Vivo 2011, 25, 757-762
1196	3292	10120	1536	cacao massLCC, LPS	In Vivo 2011, 25, 229-236
13681	1765	6598	2516	LEM	In Vivo 2010, 24, 543-552
329	5111	17109	934	Chinese herbal extracts	Anticancer Res 2009, 29, 3211-3220
		11567	1320	Mastic extracts	In Vivo 2009, 23, 215-224
56	3392	8726	1433	3-Formylchromones	In Vivo 2007, 21, 829-834
327	7179	3346	603	mulberry juice	In Vivo 2007, 21(3), 499-505
		6367	808	Trihaloacetylazulenes	Anticancer Res 2006, 26, 1917-1924
207	1487	2840	1237	Anastasia Black	Anticancer Res 2006, 25, 1991-2000
**<Mean value>**					
**13643**	**10969**	**11090**	**2300**		
**(56~137917)**	**(781~133305)**	**(2840~22893)**	**(603~11898)**		
